# 

**Published:** 1898-01

**Authors:** 


### CONTENTS.

ORIGINAL COMMUNICATIONS—

The Importance of a High Grade of Physical Health in the Effort to Cure Develop, or Reform the Dependent and Delinquent Classes Cared for in Public Institutions. By William P. Spratling, M.D., Sonyea, N. Y. .... 721
Surgery of the Gall-Bladder and Ducts. By J. McFadden Gaston, M.D., Atlanta, Ga .... 729
A Sketch of Psychiatry in the Southern States. By T. O. Powell, M.D., Milledgeville, Ga .... 736
Some Experience Gleaned from Eye and Ear Practice. By E. C. Ellett, M.D., Memphis, Tenn .... 747
A Good Spray for Nose and Throat Diseases. By George Brown, M.D., Atlanta, Ga. 759

CORRESPONDENCE—

New York Letter .... 761
Arp's Medical Reminiscences .... 766

EDITORIAL—

Defective Medical Education .... 769
Football, National Quarantine and the Governor's Veto .... 771
Meeting of the Southern Section of the American Laryngological, Rhinological and Otological Society .... 773
Announcement .... 773

MEDICAL ITEMS .... 774
BOOK REVIEWS .... 780
SELECTIONS AND ABSTRACTS .... 787
MEDICAL ITEMS—Continued .... x, xii, xiv, xvi, xvii
ADVERTISERS' INDEX TO ADVERTISEMENTS .... xviii
CLASSIFIED INDEX TO ADVERTISEMENTS .... iii

